# Interoception Dysfunction Contributes to the Negative Emotional Bias in Major Depressive Disorder

**DOI:** 10.3389/fpsyt.2022.874859

**Published:** 2022-04-11

**Authors:** Hongliang Zhou, Haowen Zou, Zhongpeng Dai, Shuai Zhao, Lingling Hua, Yi Xia, Yingling Han, Rui Yan, Hao Tang, Yinghong Huang, Yishan Du, Xiaoqin Wang, Zhijian Yao, Qing Lu

**Affiliations:** ^1^Department of Psychiatry, The Affiliated Brain Hospital of Nanjing Medical University, Nanjing, China; ^2^Nanjing Brain Hospital, Medical School of Nanjing University, Nanjing, China; ^3^School of Biological Sciences and Medical Engineering, Southeast University, Nanjing, China; ^4^Key Laboratory of Child Development and Learning Science of Ministry of Education, Nanjing, China

**Keywords:** major depressive disorders, negative emotional bias, interoception, predictive coding, HEP (heartbeat evoked potential)

## Abstract

**Background:**

Previous research studies have demonstrated that impaired interoception is involved in emotional information processing in major depressive disorder (MDD). Heartbeat-evoked potential (HEP) amplitudes, an index for interoception, could be manipulated by emotional faces in healthy people. Considering negative emotional bias is the core characteristic in MDD, we hypothesized that interoception dysfunction was associated with the negative emotional bias in MDD.

**Methods:**

An electroencephalogram (EEG) study under an emotional faces task was applied to explore the relationship between interoception and emotional bias. HEPs before emotional faces stimuli were used to predict the late positive potential (LPP) amplitudes and it worked as an index of emotional bias. Twenty-seven patients with MDD and 27 healthy controls (HCs) participated in this study. Source analysis gave an auxiliary description for results in sensory level.

**Results:**

Major depressive disorders (MDDs) had poor performance in the heartbeat count task (HCT) and attenuate HEP average amplitudes (455–550 ms). Compared with HCs, cluster-based permutation *t*-tests revealed that MDDs had attenuated LPP amplitudes (300–1,000 ms) over centroparietal regions and enhanced LPP amplitudes over frontocentral regions. Furthermore, abnormal attenuated HEPs could predict aberrant LPPs under sad face stimuli in MDDs, which could be associated with the dysfunction of the anterior cingulate cortex (ACC) and right insula.

**Conclusion:**

Mediated by ACC and insula, interoception dysfunction contributes to the negative emotional bias of MDD, highlighting the importance of interoception in the disorder.

## Introduction

Major depressive disorder (MDD) is a common affective psychiatric disorder and has been the leading cause of burden worldwide (1). Emotion dysregulation is the core characteristic of MDD and efforts to elaborate potential mechanisms of emotion are particularly pressing for the development of effective prevention and therapy in MDD (2). Previous works have reported that depressive patients are characterized by the negative emotional bias (3), concretely showing in abnormal negative faces processing (4). It was reported that depressive patients showed abnormal event-related potential (ERP) amplitude resulting from aberrant neuron discharge evoked by emotional faces stimuli (5). Compared with healthy controls (HCs), negative emotion stimuli evoked more aberrant ERPs than positive emotion stimuli and neutral emotion stimuli tended to evoke negative similar ERPs (6, 7) in depressive patients, which was mediated by the abnormal activity patterns within common networks of brain regions that include the amygdala, basal ganglia, insula, anterior cingulate cortex (ACC), and several regions of the prefrontal cortex (8–10). Late positive potential (LPP), which occurs between 300 and 1,000 ms after stimuli and maximum over centroparietal regions, is a late emotion-related ERP component specially related to negative emotional bias (11, 12). LPPs were thought to reflect emotion arousal and motivated attention for their synchronism with the arousal of the autonomic nervous system (13, 14). The attenuated mean amplitude of LPPs in MDD was associated with low dopamine levels (15).

The theory of embodied cognition indicates that not only cognitive processes are influenced by the body but also cognition exists in the service of action (16). Interoception is the core of the theory of embodied cognition, and embodied cognition supports the extension to other brain regions of the principles of organization of cerebral cortical connections (17, 18). Further investigating the relationship between interoception and depression would be helpful in understanding the neural mechanism of MDD. In psychology, predictive coding is applied to study the brain function, especially in electrophysiological investigations (19). Unlike the traditional experimental model, which recognized the brain as a “stimulus-response” organ, in the predictive coding model, the brain actively applies learned predictions to infer the causes of incoming sensory information, namely, brain could predict the following state by previous experiences in a probability-driven way (20). By integrating the neuroanatomical model, Barrett provided the Embodied Predictive Interoception Coding (EPIC) model to explain the potential pathological mechanism for MDD (21). The model speculates that the imbalance of allostasis detected by interoception dysfunction leads to diverse clinical features (such as emotion dysregulation) that result from the disabled internal-and-external information processing. ACC, insula, primary interoceptive cortex, and somatosensory regions could be the neural basis mediated by dopamine and acetylcholine (21). The neural network in this model had been proved with a large healthy sample of MRI study and macaque monkeys study, which put a new perspective to understand the emotion dysfunction in MDD (22, 23).

Up to now, many studies had revealed the correlation between peripheral system dysfunction and MDD (24–26), highlighting the important role of allostasis in MDD. It is conceived that the interoception dysfunction leads to an imbalance of allostasis in MDDs by the abnormal integration of internal and external information (27), which would affect emotion information processing ulteriorly. ERPs, with the advantages of the high temporal resolution, are beneficial to studying high informative power on neural alterations in MDD (28). Cardiac interoception working as a robust internal signal (29) is a majority way to investigate interoception because of heartbeats’ large effect and easy availability (30). By time-locking to the electrocardiogram (ECG) R-peak of the heartbeat, cardiac interoception is quantified with the heartbeat-evoked potential (HEP) (31, 32). It was reported that depressive individuals had decreased heartbeat perception accuracy and reduced HEP amplitudes (33, 34). Further evidence of an impaired association between HEP amplitudes and heartbeat perception accuracy in depressive patients suggested the imbalance of allostasis in MDD (34). In addition, many researchers had reported the relationship between emotional information processing and interoception in psychiatric disorders, such as anxiety (35), alcohol use disorder (36), and functional neurological symptom disorder (37). However, previous research studies on interoception mainly focused on somatic symptoms in MDD (27, 38). Recently, some studies have shown that HEP amplitude could be manipulated by negative emotional face stimuli in healthy people (39), which provided the clue to explore the relationship between interoception and the negative emotional bias in MDD.

The current study was aimed to study the negative emotional bias in MDD from the aspect of internal-external information integration. We supposed that there is an association between impaired interoception and negative emotional bias in MDD. We further speculate that aberrant HEPs before emotional faces stimuli could predict abnormal LPPs in MDD.

## Materials and Methods

### Ethics Statement

Procedures were approved by the ethics committee of the Affiliated Nanjing Brain Hospital of Nanjing Medical University in accordance with the Declaration of Helsinki, and all participants provided written informed consent. The data were collected from 01 March 2021 to 30 October 2021.

### Participants and Procedures

**FIGURE 1:**
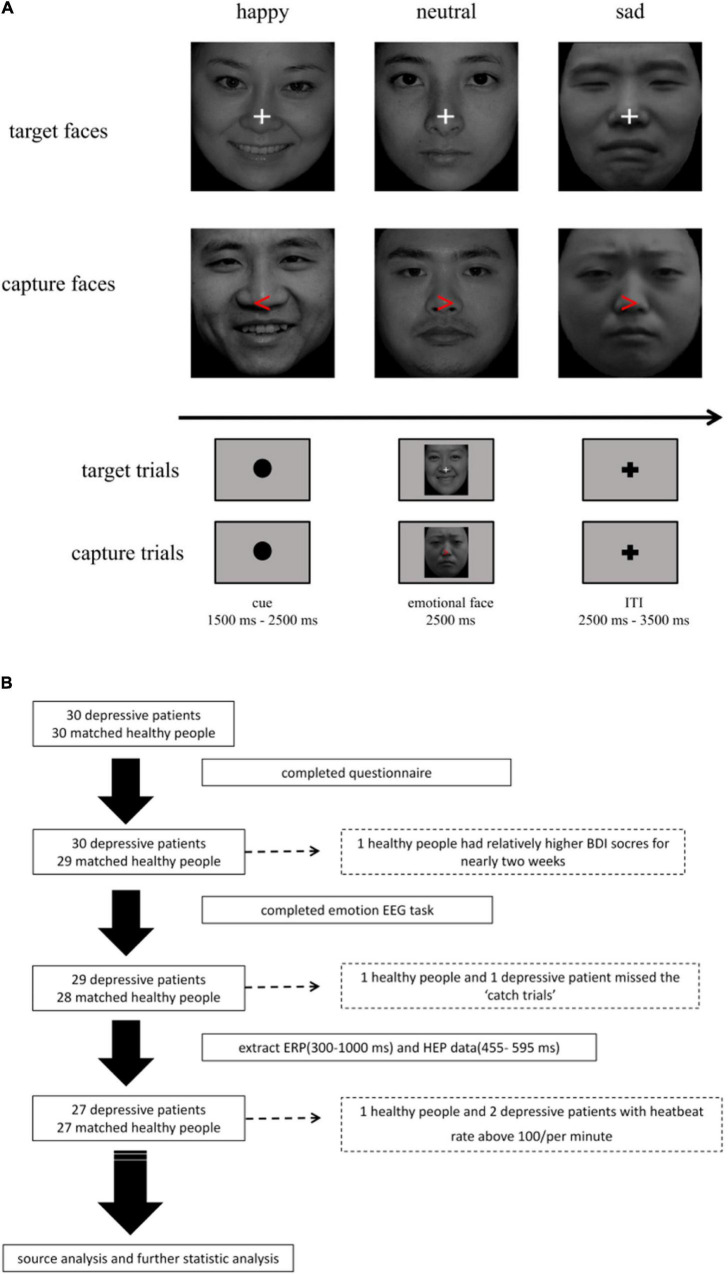
**(A)** Emotional facial stimuli task. The task was divided into two parts (target trials: 80%; capture trials: 20%). **(B)** The flowchart for the study. After processing of data, 27 major depressive disorders (MDDs) and 27 healthy controls (HCs) were involved in the task.

Participants included 30 MDDs and 30 healthy volunteers who matched for age, sex, and body mass index (BMI). The education of parents was also matched in that this information contained intelligence and economic level at the same time. MDDs fulfilled the Diagnostic and Statistical Manual of Mental Disorders Fourth Edition (DSM-IV) diagnosis of MDD as verified with the Chinese version of the Mini-International Neuropsychiatric Interview (MINI) interview to further study inclusion. Beck Depression Inventory-II (BDI-II) (40) and the State-Trait Anxiety Inventory (STAI) (41) were applied to assess the depressive level and anxiety state. General exclusion criteria comprised (a) neurological disorders; (b) alcohol dependence, nicotine dependence, or other psychoactive substance abuse; (c) comorbidity or diagnosis of other psychiatric disorders; (d) severe medical illness, such as cardiac dysregulations; (e) received medicine therapy in 2 weeks; and (f) received Electroconvulsive Therapy (ECT) or modified ECT within last 6 months. After data processing, a total of 54 participants remained (shown in Figure 1B).

After evaluating the depressive level and anxiety state, participants have a 10-min rest and completed a standard HCT (42). They were instructed to report their heartbeats numbers in a calm state during five time periods (25, 35, 45, 55, and 60 s), which were presented in random order. Guessing the number of heartbeats was forbidden. The task was performed twice and participants with large differences in reports were asked to attend the experiment another day. Heartbeat tracking score was calculated using the following formula:


$$\frac{1}{5}\sum (1-[\left(\mathrm{recorded}\mathrm{heartbeats}-\mathrm{counted}\mathrm{heartbeats}\right)÷\mathrm{recorded}\mathrm{heartbeats}])$$

Then, participants finished a revised emotional face visual paradigm.

### Produces and Task Design

An emotional face visual paradigm was used to produce emotional stimuli edited by E-prime 3.0 (Psychology Software Tools Inc.). In total, 300 gray-scale photographs of different Chinese individuals without hair posing one of three different facial expressions (100 sad faces, 100 happy faces, and 100 neutral faces, half male and half female faces in each condition) were selected from the revised version of the Chinese Facial Affective Picture System (43). All faces with white fixation crosses at the point of the nose were resized to present centrally on a computer screen at a viewing distance of 80 cm.

In the task, we set a cue stimulus with a black fixation dot ranged from 1,500 to 2,500 ms randomly in order to capture HEP before an emotional stimulus. Then, 2,500 ms emotional faces would be followed. A cue stimulus and an emotional face stimulus constituted a trial, and trials were separated by a 2,500–3,500 ms interval to avoid residual emotion effect that affected the next pre-stimulate HEP. To avoid potential confusion, we set 20% capture trials to assess participants’ attention. The capture trials contained a red arrow replacing white fixation cross randomly in the emotional faces, which need participants to make a response by pressing “A” (right) or “L” (left) as soon as possible. The participants who failed to press the button were excluded from later analyses (as shown in Figure 1A). The task was divided into three blocks. The training session contained 10 trials and the duration of each block was about 15–20 min.

### Electroencephalogram Data Acquisition

Electroencephalogram (EEG) signal was acquired from a 64-channel active electrode system with Compumedics Neuvo at a sampling rate of 1,000 Hertz (Hz), referenced to a common average reference. The electrode cap was Quik-Cap HydroCell Array made in Australia. Two additional ECG electrodes were put on the participants’ wrist and ankle on the same side. The continuous EEG signal was filtered with a 30 Hz low filter and a 0.3 Hz high filter. Considering the volume-conduction effects, the electrodes, which neared the orbital cavity, were excluded. Finally, 47 EEG channels were included in the following analysis. Independent component analysis (ICA) as implemented in EEGLAB (EEGLAB 9.0.3, University of San Diego, San Diego, CA, United States) was conducted to remove eye movements, blinks, and the cardiac field artifact (CFA) (44). The removing ICA components in every data were no more than 4. LPPs and HEPs were calculated by averaging across trials in different conditions.

### Late Positive Potential and Heartbeat-Evoked Potential Analyses

For the LPP, the segment set was put from − 200 to 1,000 ms relative to the presentation of the segment set the facial stimulus. Every participant completed 100 trials per condition and at least 70 trials (70–93 trials, average 90.47) leaving after artifact correction. The LPP in this study was defined as the mean voltage from 300 to 1,000 ms.

For the HEP, the R-peak was marked by HEPLAB [HEPLAB: a MATLAB graphical interface for the preprocessing of the HEP (Version v1.0.0)]. EEG data were segmented into 1,000–2,500 ms periods relative to the ERP stimulus’ markers to reduce the overlap from vision-evoked potential. Although in a recent study, authors were asked to segment heartbeat period with −100 to 700 ms based on the R-peak marker in the healthy population, the heartbeats of the most MDDs in our study were above average 86/per min during the task, which was in line with the authenticated phenomenon that MDDs had faster heartbeats than healthy human (45–47). It would fail to show disease attributes with segmentation standard by −100 to 700 ms. Therefore, epochs were further segmented into periods ranging from −100 to 600 ms according to the R-peak marker in our study (48–50). In HEP analysis, CFA was considered as the most important confounding factor for its same event time-locked characteristics with HEP (42). The strong electrical signal of CFA would influence results by spatio-temporal overlap with the HEP. ICA has been accepted as one of the most effective methods to excluded CFA; however, it is hard to completely remove the CFA with ICA. In our study, we had chosen the mean voltage from 455 to 595 ms after the ECG R wave as HEP index in that it was reported that CFA in this time window is less than 1% (51–54). Furthermore, the ECG mean amplitude in 455–595 ms was also compared between the two groups to avoid confounding cardiac effects (39).

Heartbeat-evoked potentials before emotion stimuli were used to predict LPPs that were induced by emotional faces.

### Source Localization of the Scalp-Domain Electrophysiological Activity

In this study, the different scalp-domain electrophysiological activity was further explored by source localization analysis, which was conducted by free standardized low-resolution brain electromagnetic tomography (sLORETA) software.^1^ The sLORETA estimates brain neural activity by solving the EEG inverse problem, which has been widely applied in the EEG source localization analysis. The time windows were set according to significant scalp-domain electrophysiological activity, and mean amplitude analysis was used to figure out the different source activation between HCs and MDDs for HEPs (455–595 ms) and LPPs (300–1,000 ms).

### Statistical Analyses

Independent *t*-tests and chi-square test were performed to compare the differences between the MDD group and HC group in demography characteristics (age, BMI, parents’ education, disease course, ECG data, and gender) and questionnaires (BDI-II, STAI) with IBM Statistical Product and Service Solution (SPSS) Statistics for Windows, version 17 (IBM Corp., Armonk, NY, United States). Non-parametric cluster-based permutation tests were performed in both sensor and source levels with a value of *p* was set for < 0.05. Partial correlation analysis was applied to examine the relationship between the significant electrodes of HEPs and LPPs, with age, BMI, gender, education of parents, disease course, BDI-II, SAI, and TAI as control variables. To eliminate the effect of dimension, data were normalized and a stepwise regression analysis was used to establish a predictive mode with the significant clusters of LPPs as the dependent variables and the significant clusters of HEPs as the independent variables. The value of *p* was set for < 0.05. The best model was selected to explain the relationship between LPP and HEP.

## Results

### Demographic and Clinical Characteristics

As shown in Table 1, there are no differences in age, BMI, gender, and the education of parents between the MDD group and HC group. However, the differences were found in heartbeat interoception, the scores of BDI-II, SAI, and TAI between the two groups.

**TABLE 1:** Demographic information for the major depressive disorder (MDD) group and healthy control (HC) group.

Variables	MDD	HC	Test statistic
Gender ratio (M/F)	27 (16/11)	27 (15/12)	χ^2^ = 0.076, *p* = 0.783
Mean age (SD)	27.0 (6.7)	25.3 (3.8)	*t* = −1.150, *p* = 0.257
BMI	21.6 (2.8)	21. 6(2.9)	*t* = −0.059, *p* = 0.953
Education (SD)	13. 6 (3.9)	14.0 (3.2)	*t* = 0.497, *p* = 0.653
Education of parents (SD)	14.7 (1.8)	15.3 (1.8)	*t* = 1.29, *p* = 0.20
Total duration of depressive episode (month, SD)	54.2 (57.8)	–	–
Number of depressive episode onset (SD)	5.1 (3.2)	–	–
BDI-II (SD)	27.9 (9.3)	2.7 (3.3)	*t* = −13.247, *p* = 0.000
SAI (SD)	54.7 (11.2)	30.3 (5.5)	*t* = −10.161, *p* = 0.000
TAI (SD)	57.7 (9.3)	30.7 (7.2)	*t* = −11.909, *p* = 0.000
HCT	0.3 (0.2)	0.5(0.2)	*t* = 2.077, *p* = 0.043
HR	70.4 (8.8)	69.9 (7.6)	*t* = −0.216, *p* = 0.830
Hit rate for catch trails	0.993(0.023)	0.994(0.016)	*t* = 0.345, *p* = 0.731

*MDD, major depressive disorder group; HC, healthy control group; F, female, M, male; SD, standard deviation; BDI-II, Beck Depression Inventory-II; SAI, State Anxiety Inventory; TAI, Trait Anxiety Inventory; HCT, heartbeat count task; HR. heartbeat rate.*

### Heartbeat-Evoked Potentials Component

**FIGURE 2:**
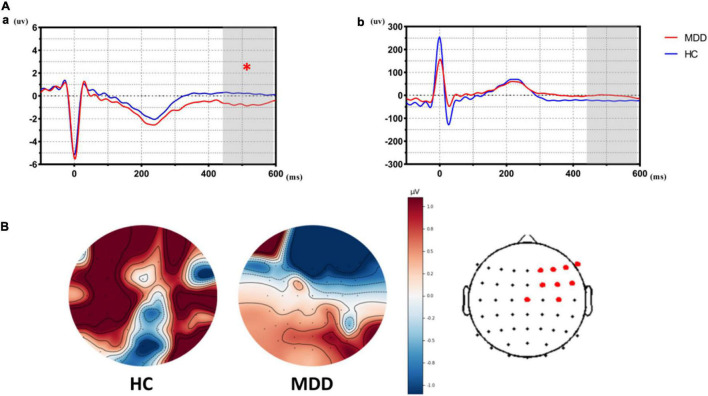
The differences of heartbeat-evoked potentials (HEPs) in the stage of affective prediction between the major depressive disorder (MDD) group and the healthy control (HC) group. **(A**a**)** Showed significant differences in HEPs during the prediction stage between the MDD group and the HC group. **(A**b**)** Suggested that significant differences did not exist in ECG during the prediction stage between the MDD group and the HC group, which indicated significant differences in HEPs did not result from the effect of cardiac field artifact (CFA). **(B)** Topological graph of HEP in both groups. Electrodes marked with red color indicate a significant group-difference.

As shown in Figure 2, HEPs during the prediction stage (predict-HEPs) are significantly attenuated in MDDs vs. HCs (*p* < 0.005, marked by the red pot in Figure 2). While there were no significant differences in ECG during the same stage, which indicated that the significant differences of HEP amplitudes were not evoked by ECG (ECG during prediction stage: *t* = − 1.291, *p* = 0.203).

### Late Positive Potentials Component

**FIGURE 3:**
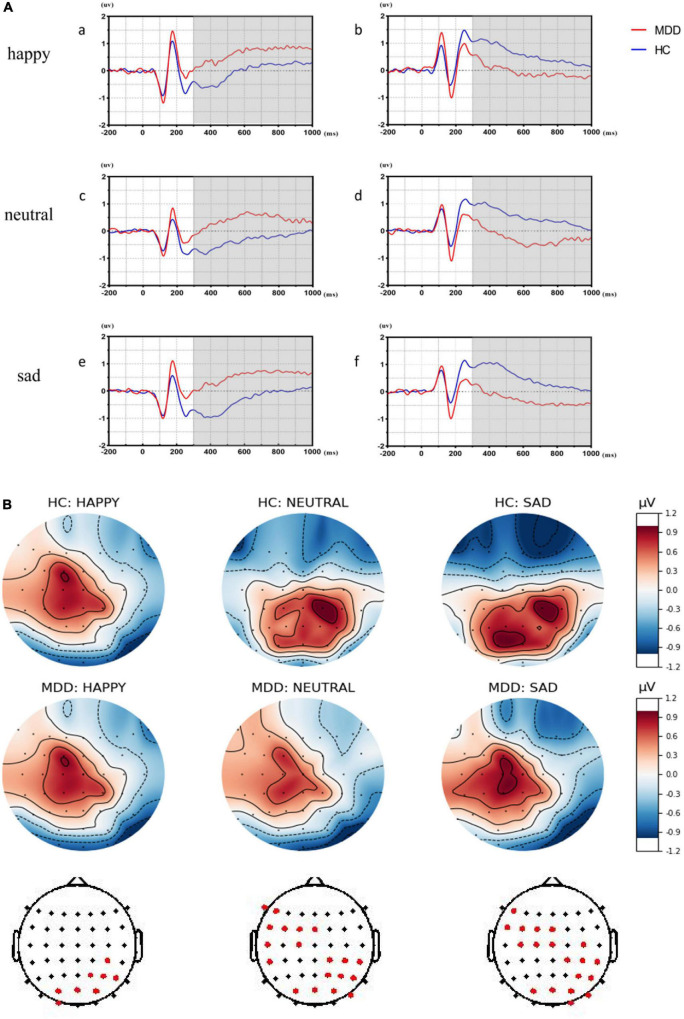
The significant differences of late positive potentials (LPPs) in different affective faces trials between major depressive disorder (MDD) group and healthy control (HC) group. **(A**a,c,e**)** Showed the significant differences over left frontocentral sites between the MDD group and the HC group by averaging significant differences in electrodes in different affective face trials. **(A**b,d,f**)** Showed the significant differences over right centroparietal sites between MDD group and HC group by averaging significant differences in electrodes in different affective face trials. **(B)** Was the top plots with significant differences for LPPs.

As shown in Figure 3, LPPs in MDDs over right centroparietal sites are significantly attenuated in all emotional face trials while LPPs over left frontocentral sites are significantly increased in sad and neutral faces than HCs (*p* < 0.005).

### Source Reconstruction of Event-Related Potential Components

Source localization analysis was applied to find the underlying responsible generators of HEPs during the prediction stage and LPPs in sad face trials. Non-parametric voxel-wise tests with *p* < 0.01 were used to compare sources between HCs and MDDs, separately for HEPs and LPPs.

### The Different Source Localization of Heartbeat-Evoked Potentials in Major Depressive Disorder Group vs. Healthy Control Group

**FIGURE 4:**
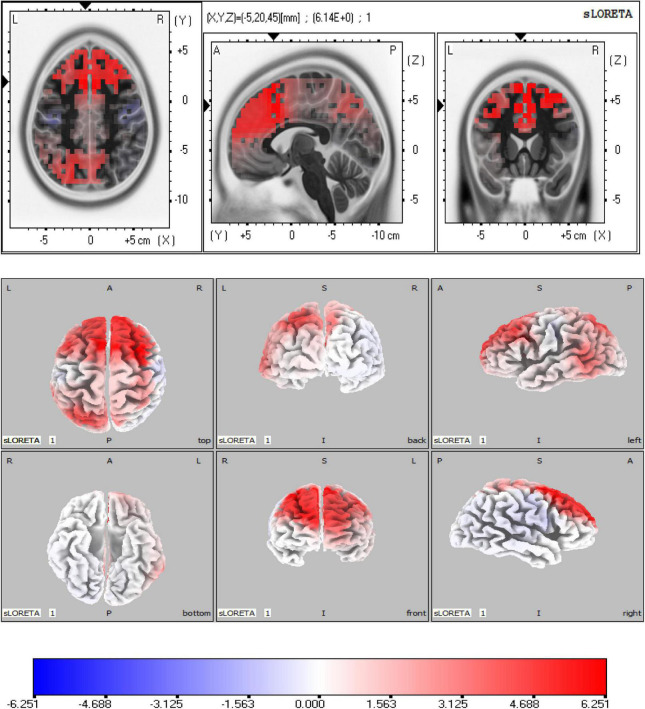
Source analysis showed the attenuated Heartbeat-evoked potentials (HEPs) in major depressive disorder (MDD) mainly resulted from the frontal lobe (BA6, BA8) and limbic lobe (BA32). The marked site was anterior cingulate cortex (ACC) (BA32).

As shown in Figure 4, the sLORETA analysis suggests that there are 963 voxels different in two groups, mainly located in the frontal lobe [middle frontal gyrus (BA6) (maximal activity at montreal neurological institute (MNI) coordinates 30, 15, and 60), superior frontal gyrus (BA8) (maximal activity at MNI coordinates 25, 20, and 55)], and limbic lobe [cingulate gyrus (BA32) (maximal activity at MNI coordinates − 5, 20, and 45)]. These significant voxels showed a higher source activation in the HC group than the MDD group.

### The Different Source Localization of Sad-Late Positive Potentials in Major Depressive Disorder vs. Healthy Control

**FIGURE 5:**
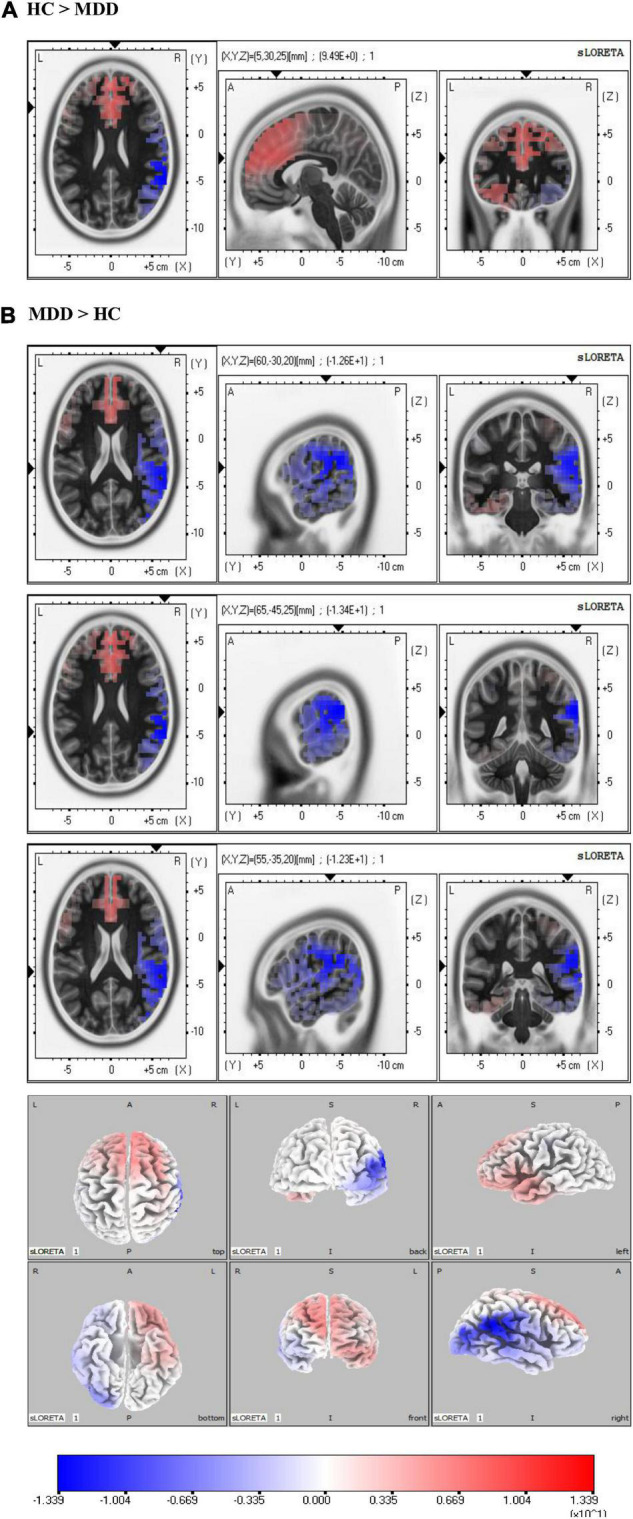
Source analysis in the time window of sad-late positive potentials (LPPs). **(A)** Revealed the activity of anterior cingulate cortex (ACC) in the healthy control (HC) group was higher than major depressive disorder (MDD) group. In **(B)**, the MDD group showed higher activity in the right parietal lobe. Especially, the right insula showed higher activity in the MDD group than the HC group.

As shown in Figure 5, the significant differences of sad-LPPs are mainly located in the right hemisphere. The sLORETA analysis showed that the source activation of the HC group was significantly higher in limbic lobe than the MDD group, especially in the anterior cingulate (BA32) (maximal activity at MNI coordinates 5, 30, and 25). While in the parietal lobe, such as the postcentral gyrus (BA2, BA40) (maximal activity at MNI coordinates 60, −30, and 20), inferior parietal lobe (BA40) (maximal activity at MNI coordinates 65, −45, 25), the source activation is higher in MDDs than HCs. In addition, the right insula (BA13) (maximal activity at MNI coordinates 55, −35, and 20) is also salient in MDDs.

### Regression Model Analysis

**FIGURE 6:**
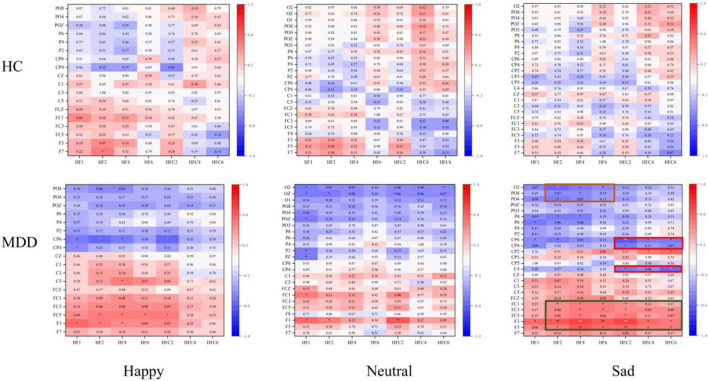
The correlation between predict-heartbeat-evoked potentials (HEPs) and sad-late positive potentials (LPPs) in major depressive disorder (MDD) and healthy control (HC) groups. The significantly correlated clusters were only found in the MDD group.

As shown in Figure 6, only under sad face stimuli, significant differences in HEPs are correlated with LPPs cluster in the MDD group. In the MDD group, HEP electrode sites have a significantly positive correlation with LPP electrode sites in three clusters as shown in Figure 4. These results showed that the all significant HEP electrode sites could positively predict LPPs in left frontocentral sites (F5, F3, FC5, FC3, and FC1) (*r* = 0.586, *p* = 0.008). HEPs in right frontocentral sites (FC2, FC4, and FC6) were negatively correlated with LPPs in centroparietal sites (C4, CP4, and CP6) (*r* = − 0.545, *p* = 0.016), while HEPs in right frontal sites (F2, F4, and F6) were negatively correlated with LPPs in parieto-occipital sites (PO4, PO8, and O2) (*r* = − 0.470, *p* = 0.042). However, the correlation did not appear in the HC group.

In addition, the item-20 in BDI-II (“evaluation of own health”) correlated with HEPs in right frontocentral sites positively in MDDs (*r* = 0.538, *p* = 0.010), while the scores of HCT were negative correlated with the scores of TAI (*r* = − 0.468, *p* = 0.028) in HCs. LPPs in right centroparietal sites (C4, CP4, and CP6) were negatively correlated with a cognitive factor of BDI-II (*r* = − 0.464, *p* = 0.034) in MDD. With stepwise regression analysis, two optimally predictive models were created. The equation is shown below:


(1) $$\mathrm{LPP}\,\left(\mathrm{F5F3FC5FC3FC1}\right)=0.091+0.540\times \mathrm{HEP}\,\left(\mathrm{F2F4F6FC2FC4FC6}\right)+0.178\times \text{gender}\;R^{2}=0.409;F=8.317$$

Where the adjusted *R*^2^ statistic was 0.360 and the *p* of the model was 0.002. The values of *p* for included variables in (1) are shown in Table 2.


(2) $$\mathrm{LPP}\,\left(\mathrm{C4CP4CP6}\right)=1.142-0.431\times \mathrm{HEP}\,\left(\mathrm{FC2FC4FC6}\right)-0.178\times \text{gender}-0.226\times \mathrm{cognitive}\,\mathrm{factor}\;{\text{R}}^{2}=0.531;\text{F}=8.696$$

**TABLE 2:** The results of the predictive model (1).

Included variables	Unstandardized coefficients (B)	Unstandardized coefficients (Std. Error)	*p*	Standardized coefficients (Beta)
HEPs (F2, F4, F6, FC2, FC4, FC6)	0.540	0.138	0.001	0.645
Gender	0.178	0.075	0.026	0.393

Where the adjusted *R*^2^ statistic was 0.470 and the *p* of the model was 0.000. The values of *p* for included variables in (2) are shown in Table 3.

**TABLE 3:** The results of the predictive model (2).

Included variables	Unstandardized coefficients (B)	Unstandardized coefficients (Std. Error)	*p*	Standardized coefficients (beta)
HEPs (FC2, FC4, FC6)	−0.431	0.121	0.002	−0.534
Gender	−0.178	0.054	0.003	−0.509
Cognitive factor	−0.226	0.110	0.052	−0.301

## Discussion

To our knowledge, this study was the first to investigate the negative emotional bias in MDD from the aspect of internal-external information integration by interoception. With converging evidence reporting the intimate connections between the peripheral nervous system and emotion in MDD (55, 56), the role of allostasis in depression pathological mechanism could not be ignored anymore. In the current study, we found that abnormal HEPs could predict aberrant LPP clusters in sad face trials only in the MDD group, indicating the possibility that aberrant interoception dysfunction has led to abnormal processing of sad faces in MDDs. Furthermore, by combing with source location analysis and previous research studies (29, 57), we cautiously provided the initial evidence that low activity of ACC might be the primary source of attenuated HEPs and predicted the overactivity of the right insula in sad face processing in MDD. These results revealed that an abnormal internal-external interactive way could be the reason for impairment of emotion processing in MDD and provide valuable clew to extend the understanding of pathological mechanism of depression.

Many studies had reported that depressive patients had abnormal interoception that is reflected by poor performance in HCT and attenuated average HEP amplitudes (27, 58). Our study had similar findings. HEP, which is generated from the cardiac signal and processed by the brain, has been proved to be a valid index for interoception (59). Previous studies focused on the relationship between HEPs and early visual evoked potentials have suggested that HEPs could predict the threshold of the visual sad faces in healthy people (60). Here, considering the relatively slow conduction of interoceptive information by weakly myelinated neurons (61), we focused on the relationship between HEPs and LPPs (62, 63). We found blunt HEPs over frontocentral regions before emotion stimulation could predict abnormal LPPs over right centroparietal sites and left frontocentral regions under emotion stimulation. More importantly, aberrant HEPs contribute to abnormal LPPs under sad emotion processing merely existed in MDD. Although limited studies in this field have been conducted in MDD, recently research studies that focused on the association between HEPs and psychiatric disorders have dramatically increased, such as social anxiety, nightmare disorder, and borderline personality disorder (57, 64–66), and revealed that interoception fluctuation contributed to perception dysfunction. Negative emotional bias seems to indicate that patients with MDD had affective perception dysfunction. Our results validated this point and further elucidated the potential neuroimaging mechanism that negative emotional bias in MDD would have resulted from the dysfunction of interoception. In our study, attenuated HEPs predicted more health-like sad-LPPs in MDD, suggesting that the HEP worked as a self-compensation to recruit more cognition resources for negative emotion processing (67–69).

Combined with source analysis, the present results showed that abnormal interoception contributed to aberrant emotion processing in two different ways in MDD. One of them was controlled by frontal electrodes. In this way, predict-HEPs could predict LPP amplitude over left frontocentral sites under sad face context, which was implicated in low activity ACC. ACC was one of the major sources of HEPs and was famous for its autonomic function (39). Generally, ACC received wide nerve projections from the frontal lobe and took part in negative emotion-related information processing (70–73). In major depression, it was reported that the connectivity between ACC and orbitofrontal cortex (OFC) was decreased with increasing depressive symptoms (29, 74) and electrical stimulation in ACC could relieve depression (75). In our study, hypoactive ACC performed its limited ability in a time frame of both predict-HEPs and sad-LPPs. Considering the crucial role of ACC in viscera signal processing and emotion processing, it was easy to understand that the low activity ACC contributed to abnormal HEPs and emotion-related LPPs, which was in consonance with the fact that HEP was correlated with LPPs over left frontocentral sites positively. The other of them was a cognition-related way that is mainly controlled by right centroparietal sites under sad content. Sad-LPPs over centroparietal sites were reported to have intimate connections with emotional motivation and were correlated with the cognition scores in BDI-II in our work. The hyperactivity of the right insula (BA40) might be the source of abnormal sad-LPPs. The right insula was famous for its high response in negative emotion processing (70, 76) and was provided to have a connection with depression’s negative emotional bias (77). Notably, the hyperactivity right insula in sadness could be predicted by hypoactive ACC, which validated the insight that interoception constitutes the emotion context by the interaction between limbic sensory (right insula) and limbic motor (ACC).

In our study, we suggested that the self-compensation of cognition source function is mediated by the information flow from ACC to the right insula. When processing sad face information, hypoactive ACC led to low emotional motivation which led to the potential negative effects, and to protect oneself, the right insula had to over-activated trying to arousing activation of ACC. This also could explain the result that blunt average amplitudes of predict-HEPs would arouse better sad-LPPs over right centroparietal sites.

In conclusion, the abnormal HEPs predict aberrant LPP amplitudes in MDD, which may result from the ACC and right insula. This study highlighted the importance of interoception for depressive patients and indicated the underlying mechanism of interoception dysfunction in emotional information processing. The study highlights interoception in MDD not only contributes to somatic symptoms but also affects emotion processing. The role of interoception in MDD is seriously undervalued. What is more, the therapy for MDD without considering homeostasis, especially at the cost of disturbing autonomic function, should be seriously reconsidered.

## Limitations

The major limitation in this study is a relatively small sample size, therefore, the results must be considered as preliminary. The cardiac rates of depressive patients were easy to increase during the task. To keep the accuracy of HEPs, we had to choose the segmentation from − 100 to 600 ms and excluded people whose heartbeats were above 86/min. In addition, considering the heartbeat is constantly changing, it is difficult and inappropriate to study the direction of information flow in our study by effective connectivity and other methods. We could only infer the process by time sequence. Future studies with larger sample sizes and optimized parameters are warranted to further verify these findings.

## Data Availability Statement

The raw data supporting the conclusions of this article will be made available by the authors, without undue reservation.

## Ethics Statement

The studies involving human participants were reviewed and approved by the Affiliated Nanjing Brain Hospital of Nanjing Medical University. The patients/participants provided their written informed consent to participate in this study.

## Author Contributions

HLZ: data curation, formal analysis, methodology, writing-original draft, and writing-review and editing. HWZ: data curation. ZPD, SZ, LLH, YX, YLH, RY, HT, YHH, YSD, and XQW: methodology. QL and ZJY: data curation, formal analysis, methodology, and writing-review and editing. All authors contributed to the article and approved the submitted version.

## Conflict of Interest

The authors declare that the research was conducted in the absence of any commercial or financial relationships that could be construed as a potential conflict of interest.

## Publisher’s Note

All claims expressed in this article are solely those of the authors and do not necessarily represent those of their affiliated organizations, or those of the publisher, the editors and the reviewers. Any product that may be evaluated in this article, or claim that may be made by its manufacturer, is not guaranteed or endorsed by the publisher.

## Footnotes

1
http://www.uzh.ch/keyinst/loretaOldy.htm
